# Rapid‐Onset Drug‐Induced Liver Injury Following Amoxicillin‐Clavulanate: A Case Report

**DOI:** 10.1002/ccr3.71655

**Published:** 2025-12-07

**Authors:** Shuvam Khadka, Abinash Dev, Pramodman Singh Yadav, Sujan Acharya, Amisha Karki, Leeza Shah

**Affiliations:** ^1^ B.P. Koirala Institute of Health Sciences Dharan Nepal; ^2^ Department of Pediatrics University of New Mexico Albuquerque New Mexico USA; ^3^ Khwopa Hospital Bhaktapur Nepal; ^4^ Department of Internal Medicine Chitwan Medical College Bharatpur Nepal

**Keywords:** amoxicillin‐clavulanate, case report, drug‐induced liver injury, hepatotoxicity, idiosyncratic reaction, RUCAM

## Abstract

Drug‐Induced Liver Injury (DILI) is a rare but potentially serious adverse effect of many commonly used medications. DILI is a diagnosis of exclusion and requires a high index of suspicion. Amoxicillin‐Clavulanate (A‐C) is a widely prescribed antibiotic known to be one of the leading causes of idiosyncratic DILI. Though well documented in Western countries, cases of DILI are less frequently reported in our region. The usual onset of DILI varies from a few days to 8 weeks after initiation of medication. Here, we report a case of an atypically rapid onset of amoxicillin–clavulanate induced liver injury in a 32‐year‐old female following just two doses.

## Key Clinical Message

This case highlights an atypical presentation of DILI. In a resource‐limited setting, high clinical suspicion and timely withdrawal of the offending medication are essential, even when confirmatory diagnostics are unavailable.

**Abbreviations**
A‐C: amoxicillin‐clavulanate
AIH: autoimmune hepatitis
ALP: alkaline phosphatase
ALT: alanine transaminase
AST: aspartate transaminase
CMV: cytomegalo virus
DILI: drug induced liver injury
EBV: Epstein‐Barr virus
RUCAM: Roussel Uclaf Causality Assessment Method

## 1 Introduction

Drug Induced Liver Injury (DILI) is an uncommon adverse drug reaction resulting in liver injury with an incidence of 14–19 cases per 100,000 individuals [1]. Among patients with acute liver failure due to DILI, the case fatality rate is 10%–15% [2]. The usual onset of DILI varies from a few days to 8 weeks after the initiation of medication [3]. DILI is a diagnosis of exclusion and requires a high index of suspicion. For likelihood assessment, the most commonly used scale is the Roussel Uclaf Causality Assessment Method (RUCAM), which provides improved reliability and reduced subjectivity in assessing suspected drug‐related injury [2, 3, 4].

Amoxicillin is a semi‐synthetic, third‐generation penicillin and clavulanate is a beta‐lactamase inhibitor. Amoxicillin‐Clavulanate (A‐C) is widely used for treating various skin, soft tissue, and respiratory tract infections. Commonly, it may cause diarrhea, nausea, and vomiting, while some potentially serious adverse effects like anaphylaxis, Stevens‐Johnson syndrome, and interstitial nephritis may rarely be seen. Even though increasing cases of idiosyncratic DILI with A‐C are reported in European and Western countries, only a few cases are reported in our part of the world [5].

This case is notable for the rapid onset of liver dysfunction shortly after two doses of A‐C, followed by rapid resolution upon discontinuation—a pattern that is unusual and sparsely documented in the literature. We present this case to highlight the importance of early clinical suspicion and recognition of atypical DILI presentations, especially in resource‐limited environments where confirmatory testing is unavailable.

## 2 Case Presentation

### 2.1 Case History/Examination

A non‐pregnant 32‐year‐old female presented to our rural Outpatient Department (OPD) having limited facilities with chief complaints of sore throat, low‐grade fever, odynophagia, and generalized weakness for the last 2 days. On examination, the patient had bilateral enlarged and erythematous tonsils with exudates. There was no icterus, lymphadenopathy, or organomegaly. No prior comorbidities, use of hepatotoxic drugs, herbal medications, or supplements were noted. The patient is a non‐smoker and non‐alcohol consumer, without any significant past, family, and travel history. The patient was diagnosed with acute tonsillo‐pharyngitis, and she was started on tablet A‐C 625 mg three times daily, tablet paracetamol 500 mg three times daily, and was suggested warm saline gargles. The next day, she returned with persistent fatigue, jaundice, and no relief in sore throat and fever, for which further lab investigations were sent.

### 2.2 Differential Diagnosis, Investigations and Treatment

Initial laboratory tests showed mild thrombocytopenia (98,000/cumm) (Ref: 150,000–400,000/cumm), with otherwise normal hemoglobin and total leukocyte counts. Monocytosis was noted on the differential leukocyte count. Renal function tests and lipid profile were within normal limits, but liver function tests were deranged. The details of her laboratory investigations, along with reference ranges, are shown in Table 1.

**TABLE 1:** Laboratory findings of the patient.

Parameter	Value	Reference range
Hemoglobin	13 g/dL	12–15 g/dL (Female)
Total leukocyte count	9650	4000–11,000/cumm
Platelet count	98,000/cumm	150,000–400,000/cumm
Monocytes (differential leucocyte count)	12	0%–8%
Total bilirubin (TSB)	4.26 mg/dL	0.3–1.4 mg/dL
Direct bilirubin (DSB)	2.53 mg/dL	0.1–0.4 mg/dL
AST (aspartate transaminase)	76 U/L	Up to 37 U/L
ALT	36.12 U/L	Up to 42 U/L
ALP	206.65 U/L	42–98 U/L (Female)
Serum creatinine	0.95 mg/dL	0.6–1.2 mg/dL
Blood urea	22.8 mg/dL	15–40 mg/dL
Random blood sugar	128 mg/dL	70–140 mg/dL
Cholesterol	172.4 mg/dL	180–200 mg/dL
Triglycerides	83.96 mg/dL	35–175 mg/dL
HDL (high‐density lipoprotein) cholesterol	71.07 mg/dL	42–88 mg/dL
LDL (low‐density lipoprotein) cholesterol	126.73 mg/dL	< 180 mg/dL

Investigations for tropical infections including dengue (Non‐Structural Protein 1 (NS1), IgM, IgG), scrub typhus (IgM, IgG), and leptospirosis (IgM/IgG) were negative. Hepatitis B surface Antigen (HBsAg) and antibody against Hepatitis C Virus (anti‐HCV antibody) were non‐reactive. Ultrasonography (USG) abdomen showed normal liver echotexture, no evidence of biliary obstruction or hepatosplenomegaly. Due to the unavailability of Epstein‐Barr Virus (EBV) and Cytomegalo Virus (CMV) serologies and Autoimmune Hepatitis (AIH) workup, these couldn't be tested. Given the temporal association with A‐C use and the exclusion of other possible causes, amoxiclav‐induced liver injury was suspected.

A‐C and paracetamol were held immediately. The patient was started on intravenous (IV) ceftriaxone and tablet azithromycin, and supportive care including hydration, warm saline gargles, and rest was advised.

### 2.3 Results (Outcome and Follow‐Up)

Following withdrawal of A‐C and paracetamol, the patient demonstrated rapid clinical and biochemical improvement. Liver enzymes and bilirubin levels decreased over the subsequent days (Table 2). Symptoms including fatigue and sore throat improved concurrently. The patient was cautioned about the use of A‐C in the future.

**TABLE 2:** Liver function test (LFT) trend.

Date	Total bilirubin (mg/dL)	Direct bilirubin (mg/dL)	ALP (U/L)	SGOT/AST (U/L)	SGPT/ALT (U/L)
Day 1	4.26	2.53	206.65	76.41	36.12
Day 2	1.80	0.70	232.76	75.00	42.0
Day 4	1.50	0.90	175.00	28.00	24.0

## 3 Discussion

DILI has an unpredictable nature and can present with a variety of clinical presentations and variable latency periods. In our case, the patient developed a cholestatic type of liver injury, depicted from an R factor value of < 2. The R factor is a calculation that is used to determine the pattern of liver injury based on liver enzyme levels [2], and it can be calculated as.

*R* = (ALT value ÷ ALT ULN) ÷ (ALP value ÷ ALP ULN), where ALT, alanine transaminase; ALP, alkaline phosphatase; ULN, upper limit of normal value. An *R* value > 5 indicates hepatocellular, < 2 indicates cholestatic, and 2–5 indicates mixed pattern.

A‐C is one of the most implicated antibiotics in idiosyncratic DILI, with a typical latency from a few days to 8 weeks. The mechanism of injury of DILI can be classified as intrinsic (dose‐dependent and occurring predictably) or idiosyncratic (unpredictable course with lack of clear dose dependence) [3]. The pattern of injury is usually cholestatic or mixed [3, 6, 7]. The precise pathogenesis of A‐C–induced liver injury remains incompletely understood and has many hypotheses. Current evidence supports an immunoallergic mechanism, while clinical features such as fever, rash, eosinophilia, and arthralgia suggest a hypersensitivity reaction [8]. Histological findings often show eosinophilic infiltration, further reinforcing this hypothesis [9]. Genetic predispositions have been noted, with strong associations reported between hepatotoxicity and Human Leucocyte Antigen (HLA) class II haplotypes, particularly DRB115:01‐DQB106:02, as well as HLA class I allele HLA‐A*02:01. Risk factors include older age, male sex, white race, and repeated exposure to the drug [9, 10, 11, 12]. Notably, clavulanate, rather than amoxicillin alone, is believed to be the primary agent responsible for the liver injury, as re‐exposure to amoxicillin without clavulanate rarely leads to recurrence [3, 13]. Even though multiple hypotheses are present, its varied presentations are not completely explained.

Our case features several atypical aspects. The patient developed symptoms within hours of drug intake, following only two doses. Moreover, the clinical course was rapid in the absence of any preexisting hepatic derangements or comorbidities, with resolution of symptoms and improvement in liver enzymes shortly after drug withdrawal. Such an early onset and rapid resolution have been rarely reported, and this case highlights the variability in latency and severity of A‐C induced liver injury.

Other differentials of liver dysfunction were considered and systematically ruled out. There was no history of alcohol intake, recent herbal supplement use, or exposure to other hepatotoxic drugs. While EBV and CMV were suspected, tests could not be performed due to resource limitations; the absence of systemic viral symptoms and rapid clinical improvement after drug withdrawal made these diagnoses less likely. AIH was considered but was unlikely given the acute nature, lack of systemic features, and spontaneous resolution. Even with such constraints, the diagnosis of DILI was determined using high clinical suspicion and was further confirmed using the RUCAM score. RUCAM remains one of the most widely accepted tools for structured assessment of DILI cases, particularly in clinical scenarios where confirmatory testing is incomplete or delayed [4]. The total RUCAM score ranges from −9 to +14, which categorizes DILI into five categories: excluded (≤ 0), unlikely (1–2), possible (3–5), probable (6–8), and highly probable (≥ 9). In this case, the possibility of diagnosis of DILI was supported by RUCAM score +5 (Table 3).

**TABLE 3:** RUCAM score (cholestatic/mixed pattern).

Criteria	Score
Time to onset (within 5 days of starting drug)	+1
Course after withdrawal (total serum bilirubin fell by more than 50% from the peak value within 180 days)	+2
Risk factors (none like alcohol use, pregnancy, 55 years of age or older)	0
Concomitant drugs (no points are given if no other medications were taken or if another agent (even a known hepatotoxin) was taken, but with an incompatible time to onset)	0
Non‐drug causes ruled out (0 points if only 4 or 5 causes in Group I are ruled out) Group I (viral hepatitis A, B and C, biliary obstruction, alcoholic liver disease, and ischemic hepatitis) and Group II (complications of underlying diseases or conditions)	0
Known hepatotoxicity of the drug	+2
Rechallenge (not done)	0
Total	+5 (possible)

This case underscores several important clinical implications. As A‐C is a widely used antibiotic and is rampantly prescribed in our part of the world, it reiterates the need for clinicians to maintain a high index of suspicion for DILI even when drug exposure appears minimal or recent. Secondly, it illustrates the importance of promptly discontinuing the suspected agent, which in many cases can lead to rapid resolution without the need for invasive testing. Finally, it reflects the diagnostic challenges posed in resource‐limited settings, where comprehensive laboratory evaluation may not be feasible, emphasizing the value of detailed clinical history and structured causality tools like RUCAM.

While this case adds valuable insight, certain limitations should be acknowledged. The lack of viral serologies and autoimmune markers reduces the diagnostic certainty, although the clinical course strongly favors drug‐induced injury.

## 4 Conclusion

This case adds to the growing recognition of atypical DILI presentations and highlights the importance of considering drug‐induced injury even after minimal exposure to commonly prescribed antibiotics, especially in resource‐limited environments where confirmatory testing is unavailable. Prompt recognition and drug withdrawal remain key in ensuring favorable outcomes.

## Author Contributions

**Shuvam Khadka:** conceptualization, formal analysis, project administration, visualization, writing – original draft. **Abinash Dev:** supervision, validation, writing – review and editing. **Pramodman Singh Yadav:** data curation, supervision, visualization, writing – review and editing. **Sujan Acharya:** conceptualization, investigation, resources. **Amisha Karki:** data curation, methodology, project administration. **Leeza Shah:** methodology, supervision, validation, writing – review and editing.

## Funding

The authors have nothing to report.

## Disclosure

The patient expressed concern about the severity of her symptoms after taking just two antibiotic doses. She appreciated the explanation provided and has been compliant with follow‐up.

## Consent

Written informed consent was obtained from the patient for publication of this case report.

## Conflicts of Interest

The authors declare no conflicts of interest.

## Data Availability

All relevant data supporting the conclusions of this case report are included within the article. No additional datasets were generated or analyzed during the current study.
